# Three-dimensional paper-based slip device for one-step point-of-care testing

**DOI:** 10.1038/srep25710

**Published:** 2016-05-13

**Authors:** Kwi Nam Han, Jong-Soon Choi, Joseph Kwon

**Affiliations:** 1 Biological Disaster Analysis Group, Korea Basic Science Institute, Daejeon 169-148, Korea

## Abstract

In this study, we developed a new type of paper-based analytical device (PAD), the three-dimensional (3D) slip-PAD, to detect infectious human norovirus for global healthcare. The 3D configuration of the papers combined with a slip design provides unique features and versatility that overcome the limitations of fluidic manipulation and sensitivity in point-of-care (POC) tests. The assay can be carried out in a single step based on a moveable slip design, making it suitable for unskilled users. The 3D fluidic network developed by layered construction of wax-patterned papers provides different fluidic paths for the sequential delivery of multiple fluids without the need for peripheral equipment. The release and mixing of enhancement reagents on the device improved the sensitivity and detection limit. The assay results could be visualized by naked eye within 10 min, with subsequent amplification of the signal over time (<60 min). The device showed a broad dynamic range of detection and high sensitivity, with a detection limit of 9.5 × 10^4^ copies ml^−1^ for human norovirus. These results demonstrate that the 3D slip-PAD is a sensitive diagnostic assay for detecting human norovirus infection that is particularly suitable for POC testing in regions where resources are scarce.

There is an increasing demand for point-of-care (POC) tests in many fields, including clinical analysis, food safety, and environmental assessment, and many efforts have been made to develop biosensors that are affordable, sensitive, specific, user-friendly, rapid and robust, equipment-free, and deliverable to end-users (ASSURED) that meet World Health Organization criteria^1^,^2^,^3^,^4^,^5^. Paper-based analytical devices (PADs) have received considerable attention for POC applications owing to the advantages of low cost, light weight, ease of handling (e.g., folding, cutting, patterning, etc.), and fluid transport by capillary wicking^6^,^7^,^8^. In particular, a lateral flow assay (LFA) based on PADs is commercially available for a variety of applications that is low cost, rapid, simple to use, equipment-free and easily mass produced, thus conforming to ASSURED criteria^9^. However, it also has limitations in terms of sensitivity and manipulation of fluid flow.

To overcome these drawbacks, recent research on PADs has focused on improving detection signal and modifying paper geometry. For example, various detection labels [nanoparticles (NPs), carbon nanotube, quantum dots, fluorescent dyes, etc.] and amplification methods (enzymatic reaction and metallic precipitation) have been used to improve detection signal and sensitivity^10^,^11^,^12^,^13^. However, these do not meet the growing demand in sensitivity, and require multiple amplification steps (successive addition of reagents and washing) for optical or electrochemical detection. On the other hand, PADs with different types of geometry [three-dimensional (3D), folding, and slip, etc.] have been developed to manipulate fluidic flow and to retain operational simplicity^14^,^15^,^16^,^17^,^18^,^19^,^20^. A 1D lateral flow can be extended to 3D flow by assembling papers with hydrophilic/hydrophobic channels, which enables the control of liquid flow in a 3D fluidic network. Nonetheless, developing a one-step device that affords sequential delivery of multiple fluids for detection in a single step remains a challenge.

Norovirus is a highly infectious pathogen that is the major cause of nonbacterial epidemic outbreaks of gastroenteritis around the world^21^. According to the Centers for Disease Control and Prevention, norovirus causes 19–21 million infections, resulting in 56,000–71,000 hospitalizations and 570–800 deaths each year in the United States. The virus can be transmitted by ingestion of contaminated food or water, person-to-person contact, and contact with contaminated surfaces or objects. There are currently no vaccines or antiviral drugs for the treatment of norovirus infection; therefore, a rapid and sensitive detection system is essential for preventing and reducing norovirus outbreaks in both developed and developing countries. There are several methods for detecting norovirus in humans, including electron microscopy, enzyme-linked immunosorbent assay (ELISA), real-time reverse transcription (RT-)-PCR, and immunochromatographic LFA^22^. Although RT-PCR is widely employed owing to its high sensitivity relative to other methods, it requires expensive, sophisticated equipment and trained personnel as well as time-consuming preparation steps, making it unsuitable for POC testing. In contrast, LFA shows considerable potential as a POC device for norovirus detection based on its advantages of rapidity, simplicity, and low cost, although it is limited by insufficient sensitivity, as previously stated.

To this end, we developed a novel one-step 3D slip-PAD for simple, sensitive, and inexpensive detection of human noroviruses. A 3D fluidic network developed by layered construction of wax-patterned papers provided the difference in fluidic paths for the sequential delivery of multiple fluids without peripheral equipment. The integration of multiple reagents for signal amplification enhanced sensitivity and improved the limit of detection (LOD). Moreover, the manually movable slip design of the 3D slip-PAD enabled the one-step assay without any additional manipulation, making it more accessible to an unskilled user and therefore more suitable for resource-limited settings.

## Results

**Figure 1:**
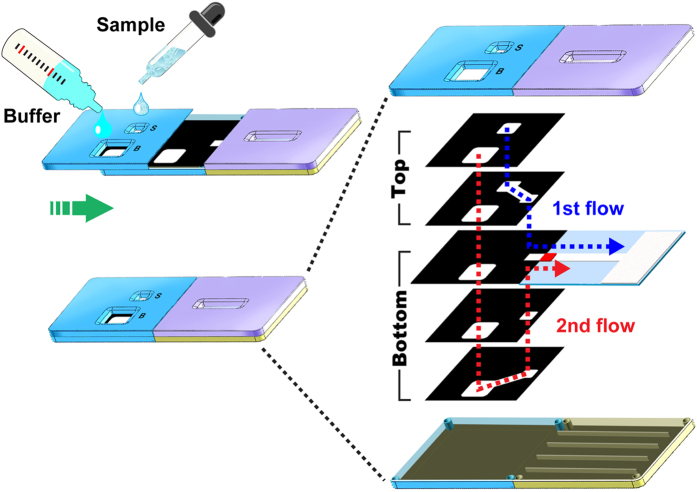
Schematic representation of the 3D slip-PAD and its operating principles for sequential fluidic manipulation. By sliding the slip-top section to the right, fluids are wicked onto adjacent paper layers, leading to the sequential delivery of fluids to the detection zone via different 3D fluidic paths. Black and white regions represent the hydrophobic wax barrier and hydrophilic fluidic channel, respectively.

The configuration of the 3D slip-PAD and its operating principle for fluidic manipulation are shown in Fig. 1. The device consisted of slip-top and stationary bottom sections in a plastic casing. The slip top was composed of two paper layers, and the bottom comprised three paper layers as well as a conjugate pad, nitrocellulose membrane, and absorbent pad arranged from left to right. The sample and buffer (or reagent) were added to the fluidic inlets and the slip-top section was slid aside to start the assay; then, the fluids began to spread to adjacent paper layers with which they were in contact. The perpendicular overlap of the multiple paper layers in the two sections provided a 3D fluidic channel for delivery of fluids to the detection zone (the design of paper layers is shown in Supplementary Figure S1).

**Figure 2:**
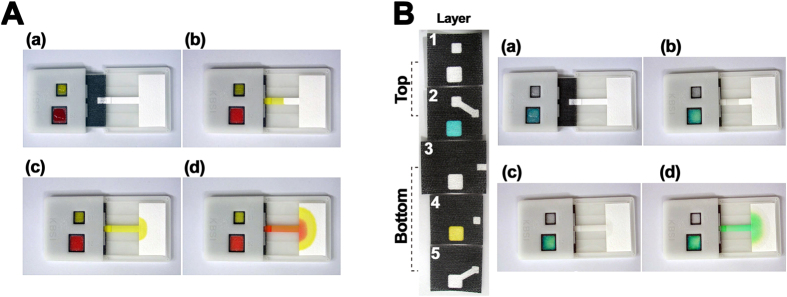
(**A**) Sequential delivery of different fluids in the 3D slip-PAD. The yellow dye first reached the detection zone, followed by the red dye. (**B**) Release and mixing of integrated reagents on multiple layers. Blue and yellow dyes were pre-integrated by drop-drying on layers 2 and 4, respectively; the mixing of the two dyes yielded a green color on the device.

The flow of different fluids on the 3D slip-PAD is illustrated in Fig. 2A. Yellow (tartrazine) and red (Allura Red) food dyes were applied to the slip-top section instead of samples and reagents so as to visualize fluidic flow. The movable slip section acted as a switch for capillary wicking: off/on switching was achieved by sliding this section to the left or right, respectively. When switched off, the impermeable wax barrier patterned on the paper layers prevented fluid from spreading onto adjacent paper layers of the stationary bottom section. To operate the device, we added 110 and 200 μl of yellow and red dye, respectively, to the fluidic inlets of the slip section [(a) in Fig. 2A]. When the slip section was manually moved to the right (switched on), the two different dyes wicked into the 3D fluidic paths of the wax-patterned paper layers, resulting in the sequential flow of the different fluids on the device [(b–d) in Fig. 2A and Supplementary Movie S1].

The release and mixing of integrated reagents on multiple layers is demonstrated in Fig. 2B. This is an important feature of POC testing devices used in resource-limited settings, since most signal transduction and amplification methods rely on chemical reactions. In a preliminary study, two different dyes were used instead of multiple reagents for signal amplification based on enzymatic or metallic reactions that are commonly used in optical/electrochemical detection strategies. Blue (Brilliant Blue FCF) and yellow food dyes were drop-dried on a single inlet of layers 2 and 4, respectively. DI water was added to two fluidic inlets [(a) in Fig. 2B], and the slip section was moved to the right. Sequential fluidic flow was carried out in the same manner as shown in Fig. 2A. Meanwhile, the pre-dried blue and yellow dyes were released and mixed in the stream of fluid, producing a green color on the device [(d) in Fig. 2B and Supplementary Movie S2]. These results indicated that the device can effectively integrate different reagents.

**Figure 3:**
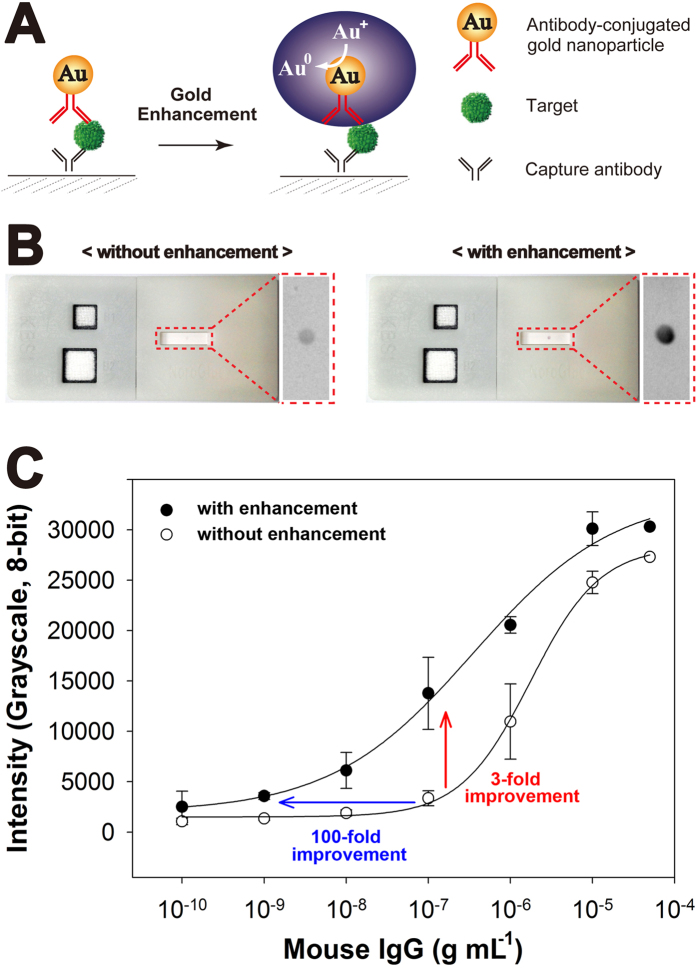
(**A**) Schematic representation of gold enhancement used for signal amplification on the 3D slip-PAD. Enlarged gold particles regulated in an enhancement of signal intensity. (**B**) Photomicrographs of the 3D slip-PAD obtained with/without gold enhancement at a target IgG concentration of 500 ng ml^−1^. (C) Binding curves obtained with/without signal enhancement; target IgG concentration varied from 0.1 ng ml^−1^ to 50 μg ml^−1^ and samples were tested in triplicate.

As a proof-of-concept demonstration for POC applications, an immunochromatographic assay coupled with a signal amplification method was implemented, using mouse IgG as a target. Capture antibody specific to the target was pre-immobilized on a nitrocellulose membrane, and signal probe (AuNP)-labeled detection antibody was drop-dried on the conjugate pad. Four different enhancement reagents were pre-integrated on a single inlet of layers 1–4. After adding 110 μl of target sample and 200 μl of DI water to each inlet of the slip-top section, the one-step assay was initiated by moving the slip top to the side. When the sample reached the conjugate pad, it dissolved AuNP-labeled antibody conjugates, forming target/labeled detection antibody complexes that continued to flow along the paper until they bound to the capture antibody on the nitrocellulose membrane according to the principle of the sandwich assay. This resulted in the appearance of color in the detection zone from the accumulation of AuNPs, indicating the presence of target in the sample. The mixture of released enhancement reagents subsequently flowed into the detection zone of the device, allowing more distinct visualization of AuNPs. Gold enhancement follows a similar autometallographic process to conventional silver enhancement in signal amplification^23^,^24^. AuNPs functionalized with antibody act as the seed or nucleation site for the specific deposition of gold, which reduces activation energy and increases reaction rate. Au ions in the mixture were catalytically reduced around the surface of AuNPs, thereby increasing their size and affording better visualization over an expanded dynamic range (Fig. 3A).

Results for IgG detection obtained with/without signal enhancement at the same target concentration of 500 ng ml^−1^ are shown in Fig. 3B. The original signals were intensified after amplification. The clear difference in signal intensity between the two results revealed that the signal was effectively enhanced on the device. This indicates that the 3D slip-PAD can provide sequential fluidic flow and release/mixing of integrated reagents, enabling highly sensitive analysis with operational simplicity. Moreover, the sensitivity and LOD were further improved by controlling assay time (Supplementary Figure S2); the efficiency of signal enhancement was monitored by the ratio of signal intensities of the amplified to non-amplified signal (I/I_0_). The enhancement ratio gradually increased over time before reaching a plateau after 60 min. Therefore, in subsequent experiments, we measured assay results after 60 min. Detection curves obtained with/without signal enhancement are shown in Fig. 3C. For a quantitative comparison of the two curves, we measured a 10-fold dilution series of target mouse IgG ranging from 0.1 ng ml^−1^ to 50 μg ml^−1^ in triplicate experiments. Data in both plots were obtained from captured images by averaging grayscale values using ImageJ software (Supplementary Figure S3). The 3D slip-PAD coupled with signal enhancement provided a >3-fold improvement in signal detection as compared to a conventional method without amplification. Moreover, the dynamic range of detection expanded by >100-fold, which is important for accurate quantification.

**Figure 4:**
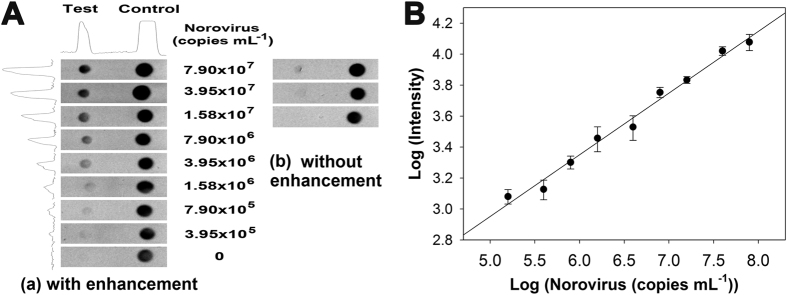
(**A**) Capture images of detection zone on the 3D slip-PAD at human norovirus concentrations ranging from 1.58 × 10^5^ to 7.9 × 10^7^ copies ml^−1^. (**B**) Log-log plot of grayscale intensity vs. norovirus concentrations corresponding to (a). Error bars represent standard errors from three independent experiments.

We demonstrated the practical application of the 3D slip-PAD in detecting human norovirus for use in POC testing. The GII.4 norovirus was chosen as the target since the GII.4 genotype is known as the major cause of norovirus infections responsible for epidemics of gastroenteritis worldwide^21^. Human fecal sample with known viral copy numbers (1.58 × 10^8^ copies ml^−1^) determined by real-time PCR was tested in Fig. 4. Fecal samples were prepared as serial dilutions in test buffer (0.01% SDS in PBS). Figure 4A shows assay results at concentrations of human norovirus ranging from 1.58 × 10^5^ to 7.9 × 10^7^ copies ml^−1^. The control zone in which secondary capture antibody was pre-immobilized ensured the validity of test; color in the control zone indicated that the test was valid. As expected, there was a marked difference in signals obtained with and without enhancement (Fig. 4A); the corresponding detection curves showed improved detection intensity (around 5 orders of magnitude) and expanded dynamic range (around 100 orders of magnitude) after signal enhancement (Supplementary Figure S4). Log-log plots corresponding to the test zone with enhancement showed a good linear relationship between log values of signal intensity and norovirus target concentration, with a correlation coefficient of 0.986 (Fig. 4B). LOD (3 SD) and quantitation limit (LOQ, 10 SD) were estimated to be 9.5 × 10^4^ and 6.3 × 10^5^ copies ml^−1^, respectively. Comparing these results to those reported in previous studies, it was found that the LOD value was up to 100-fold lower than that obtained by microfluidic RT-PCR (1.6 × 10^7^)^25^, ELISA (4.2 × 10^8^)^26^, and commercial or developed LFA kits (10^6^–10^7^)^27^,^28^,^29^. In addition, the 3D slip-PAD retained >90% of the initial signal value after 2 weeks when stored at 4 °C under dry conditions (data not shown).

## Discussion

We develop a one-step 3D slip-PAD that embodies features desirable in a POC testing biosensor, including low cost, sensitivity, robustness, rapidity, and ease of use. The device was fabricated by assembling wax-patterned paper layers and was successfully applied to the detection of human norovirus based on immunochromatography coupled with signal amplification. The device showed a wide dynamic range of detection and high sensitivity, with a LOD of 9.5 × 10^4^ copies ml^−1^ for GII.4 norovirus, which was far superior to conventional LFA kits (~10^7^ copies ml^−1^). The unique configuration of the device enabled the sequential delivery of multiple fluids without the need for peripheral equipment, as well as the release and mixing of integrated reagents for signal amplification in a single step, making it suitable for POC testing in resource-limited settings. Moreover, the format of the 3D slip-PAD is amenable to various types of optical/electrochemical assay by modifying the paper configuration or changing the integrated reagents. Based on these features and its versatility, we suggest that the 3D slip-PAD will serve as a new diagnostic tool in the fields of medicine, food/water safety, veterinary medicine, and environmental and agricultural testing.

## Methods

### Materials

Materials used for components of the 3D slip-PAD were as follows: conjugate pad (fusion 5) and cellulose absorbent pad (CF4) (GE Healthcare, Piscataway, NJ, USA); nitrocellulose membrane (Hi-Flow Plus HF120; Millipore, Billerica, MA, USA); cellulose/polyester paper (Seoul Semitech, Hwaseong, South Korea); and adhesive polyester film (Bio Rad, Hercules, CA, USA). For the preliminary study, goat anti-mouse IgG (capture and detection antibody), rabbit anti-goat IgG (control capture antibody), and mouse IgG (target) were purchased from Sigma-Aldrich (St. Louis, MO, USA). Two antibodies specific to human norovirus (mouse anti-norovirus GII.4 antibody for capture and rabbit anti-norovirus capsid protein VP1 antibody for detection) as well as goat anti-rabbit IgG (control capture antibody) were obtained from Abcam (Cambridge, UK) and Sigma-Aldrich, respectively. Human fecal samples containing NoV GII-4 subtype were obtained from Gwangju Health and Environment Research Institute. Sodium dodecyl sulfate (SDS) was from Biosesang Inc. (Seongnam, South Korea); bovine serum albumin (BSA) was from BioShop Canada Inc. (Burlington, ON, Canada); colloidal gold (Au)NPs (40 nm) were from Median Diagnostics Inc. (Chuncheon, South Korea); and the gold enhancement reagent kit was purchased from Nanoprobes Inc. (Yaphank, NY, USA). All other chemicals were of analytical grade. Aqueous solutions were prepared with deionized (DI) water.

### Preparation of detection antibody/AuNP conjugate

To optimize the conjugation of antibody to AuNPs, a series of AuNP solutions with different pH values was prepared using 0.1 M NaOH. A 3-μl volume of antibody solution (0.5–1 mg ml^−1^) was added to 100 μl AuNP solution with pH values ranging from 6 to 9. After 5 min, 20 μl of 10% NaCl was added, followed by a 2-h incubation at room temperature. NaCl causes the aggregation of AuNPs and a color change from red to purple; the optimal pH value was determined as the minimum pH without color change. Different volumes (2–20 μl) of antibody solution (0.1 mg ml^−1^) were added to 100 μl of AuNP solution at the optimal pH, followed by 20 μl of 10% NaCl. After a 2-h incubation, the optimal amount of antibody for the antibody/AuNP conjugate was selected as described above. Based on these conditions, 1 ml AuNP (pH 7.5) solution was mixed with 10 μl antibody (0.5–1 mg ml^−1^) and maintained at room temperature with gentle stirring. After 2 h, 50 μl of 10% BSA in phosphate-buffered saline (PBS) were added for 30 min to block unreacted sites on the AuNP surface. The mixture was centrifuged at 6,500 rpm for 25 min; the supernatant was discarded and the pellet was resuspended in 75 μl PBS containing 0.01% Tween 20 and 1% BSA, and stored at 4 °C for further experiments.

### Preparation of 3D slip-PAD components

Layers of patterned cellulose/polyester paper were prepared using a commercially available wax printer (Color Cube 8870, Xerox, Norwalk, CT, USA). The patterns for each layer are shown in Supplementary Figure S1. The paper was heated at 100 °C for 1 min in the dry oven to melt the patterned wax. The patterned paper layers were cut and treated with blocking agent (2% BSA in PBS) for 30 min. For the integration of enhancement reagents, a 80-μl volume of each of four reagents in an enhancement kit was drop-dried on a single inlet (1.2 × 1.2 cm) of each paper layer. The conjugate pad (0.5 × 0.5 cm) was prepared by drop-drying 10 μl of detection antibody/AuNP conjugate. For the detection region, equal volumes (0.25 μl) of capture antibody (1 mg ml^−1^) and control capture antibody (1 mg ml^−1^) were spotted on the nitrocellulose membrane (2.5 × 0.5 cm), with 5-mm spacing between them. The membrane was blocked with 2% BSA for 5 min.

### Assembly of the 3D slip-PAD

The device consisted of slip-top and stationary bottom sections in a plastic casing (10.7 × 5.0 cm). The slip top was composed of two paper layers; the stationary bottom comprised three paper layers and the conjugate pad, nitrocellulose membrane, and absorbent pad. The paper layers of the slip and bottom sections were glued together by aligning the paper edges. The aligned bottom layers, conjugate pad, nitrocellulose membrane, and absorbent pad (2.2 × 4.0 cm) were then placed in sequence on an adhesive polyester film with ~1 mm of overlap, and the slip and bottom sections were inserted in the plastic casing (Supplementary Figure S5).

### Assay procedure and signal quantification

The 3D slip-PAD-based assay required only a single step. A 110-μl volume of sample and 200 μl DI water were applied to each inlet of the slip-top section, which was moved aside to initiate the assay. The results were visible by naked eye within 10 min, with the signal becoming amplified over time (<60 min). Therefore, for maximum signal intensity, the assay was completed in 60 min. To quantify the results, ImageQuant LAS 4000 (GE Healthcare) was used to capture images of the paper with 8-bit grayscale settings. ImageJ software (National Institutes of Health, Bethesda, MD, USA) was used to analyze signal intensity in the detection zone.

## Additional Information

**How to cite this article**: Han, K. N. *et al*. Three-dimensional paper-based slip device for one-step point-of-care testing. *Sci. Rep.* **6**, 25710; doi: 10.1038/srep25710 (2016).

## Supplementary Material

Supplementary Information

Supplementary Movie S1

Supplementary Movie S2
