# Supplementary material for: Three-dimensional paper-based slip device for one-step point-of-care testing
Source: Sci Rep. 2016 May 13;6:25710. doi: 10.1038/srep25710 (PMC4865726; doi:10.1038/srep25710)
Supplement: Supplementary Information [file srep25710-s1.doc]

**Three-dimensional paper-based slip device for one-step point-of-care testing**

Kwi Nam Han, Jong-Soon Choi and Joseph Kwon

Biological Disaster Analysis Group, Korea Basic Science Institute, Daejeon 169-148, Korea

Corresponding authors:

Tel.: +82 42 865 3446

E-mail: joseph@kbsi.re.kr (J.K.) and jschoi@kbsi.re.kr (J.-S.C.)

**Supplementary Information**


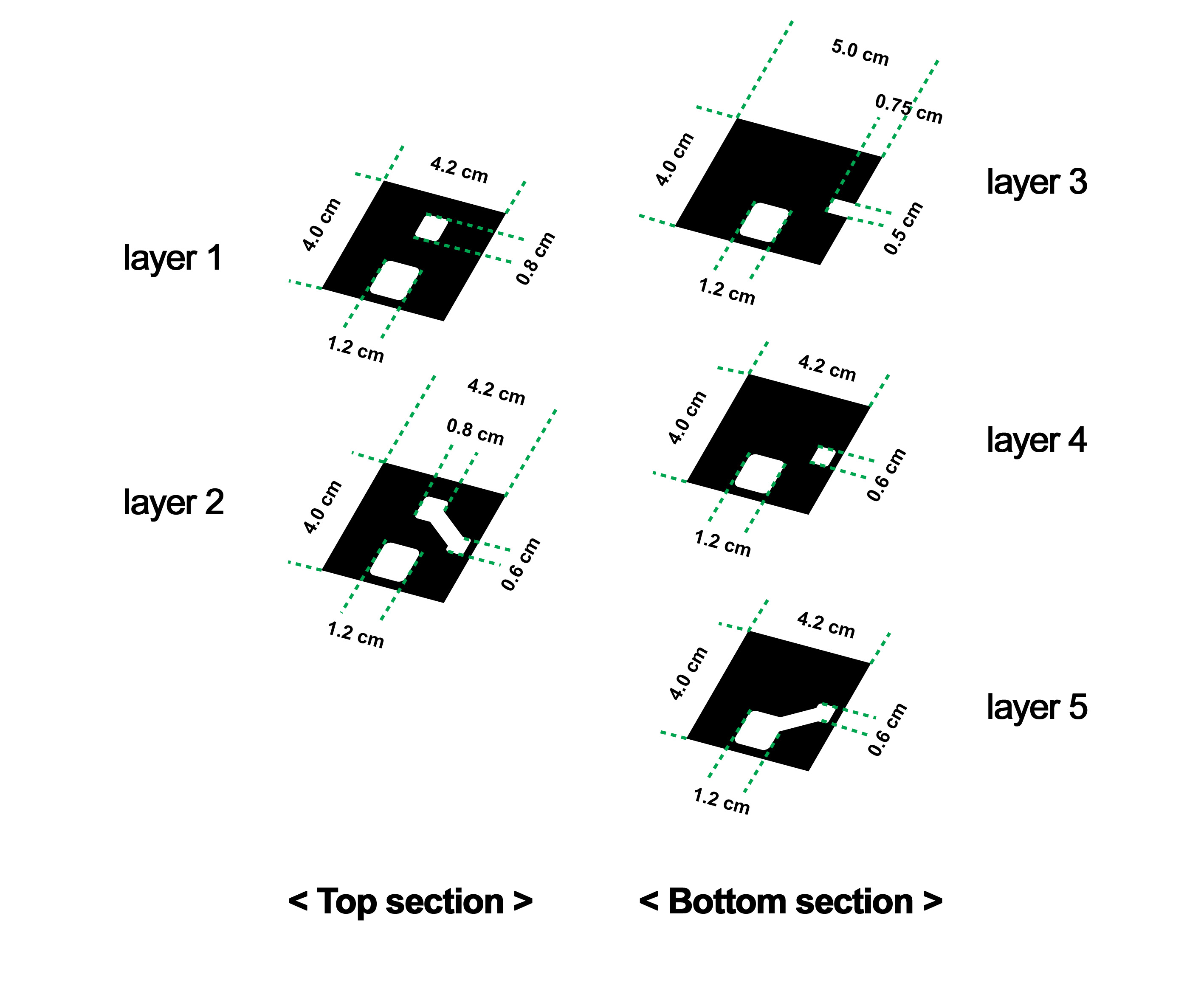


**Figure S1.** Design of internal paper layers for the construction of the 3D fluidic path.


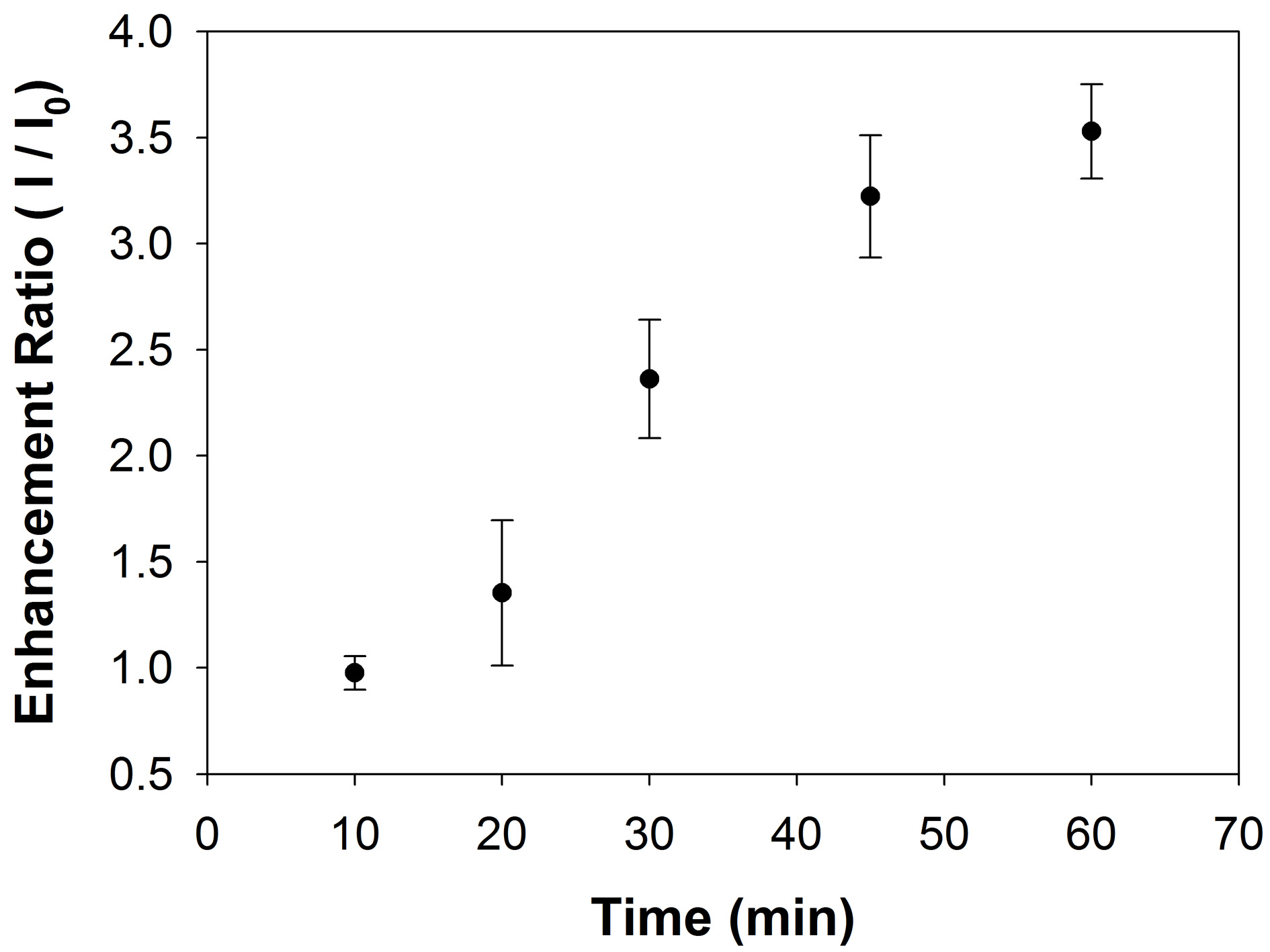


**Figure S2.** Effect of assay time on signal amplification. Target IgG concentration was maintained at 100 ng ml^−1^. The enhancement ratio (I/I_0_) was equal to amplified intensity divided by non-amplified intensity. The Au enhancement time varied from 10 to 60 min.


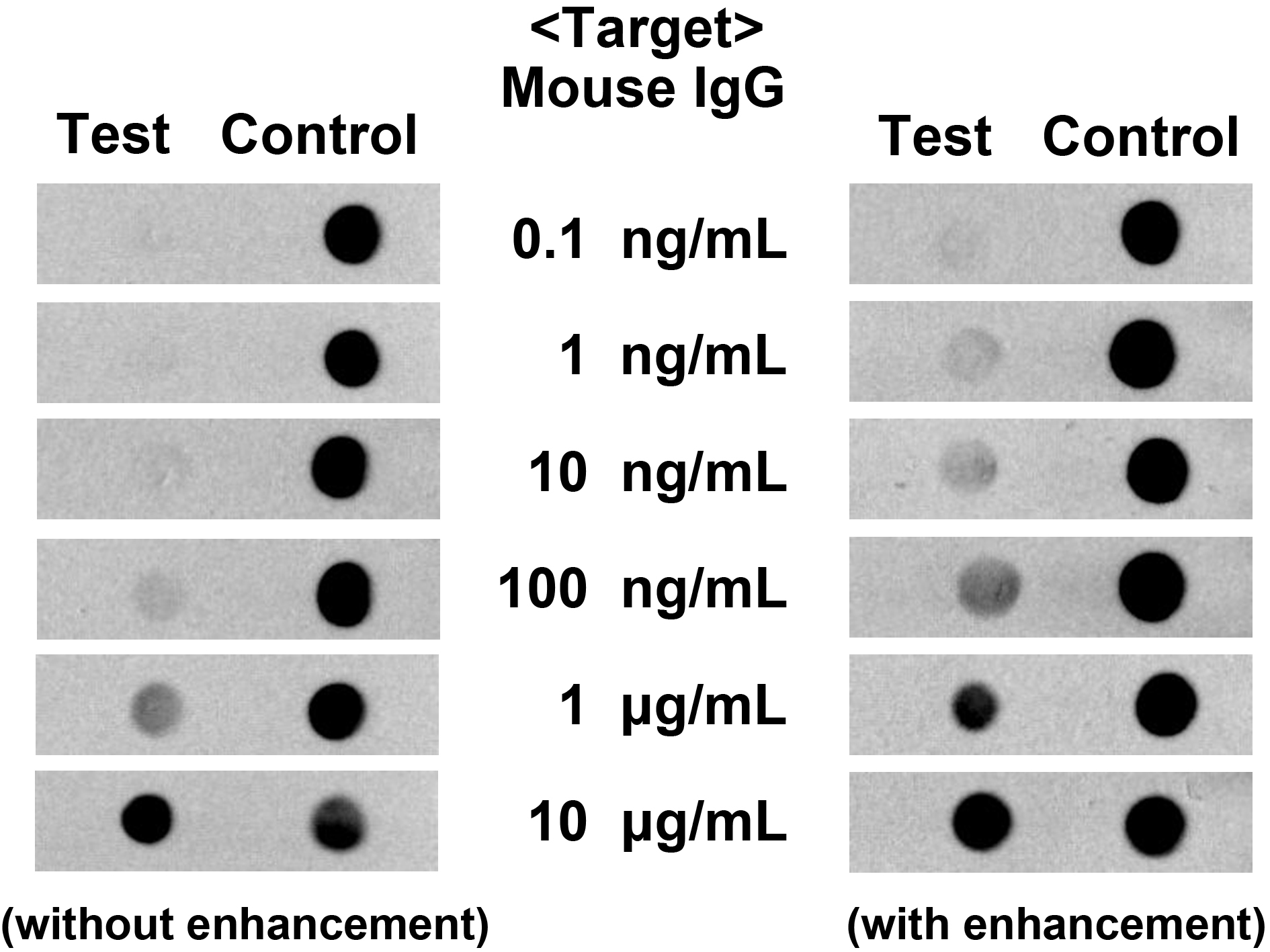


**Figure S3.** Capture images of the detection zone obtained with/without signal enhancement at indicated concentration of target mouse IgG.


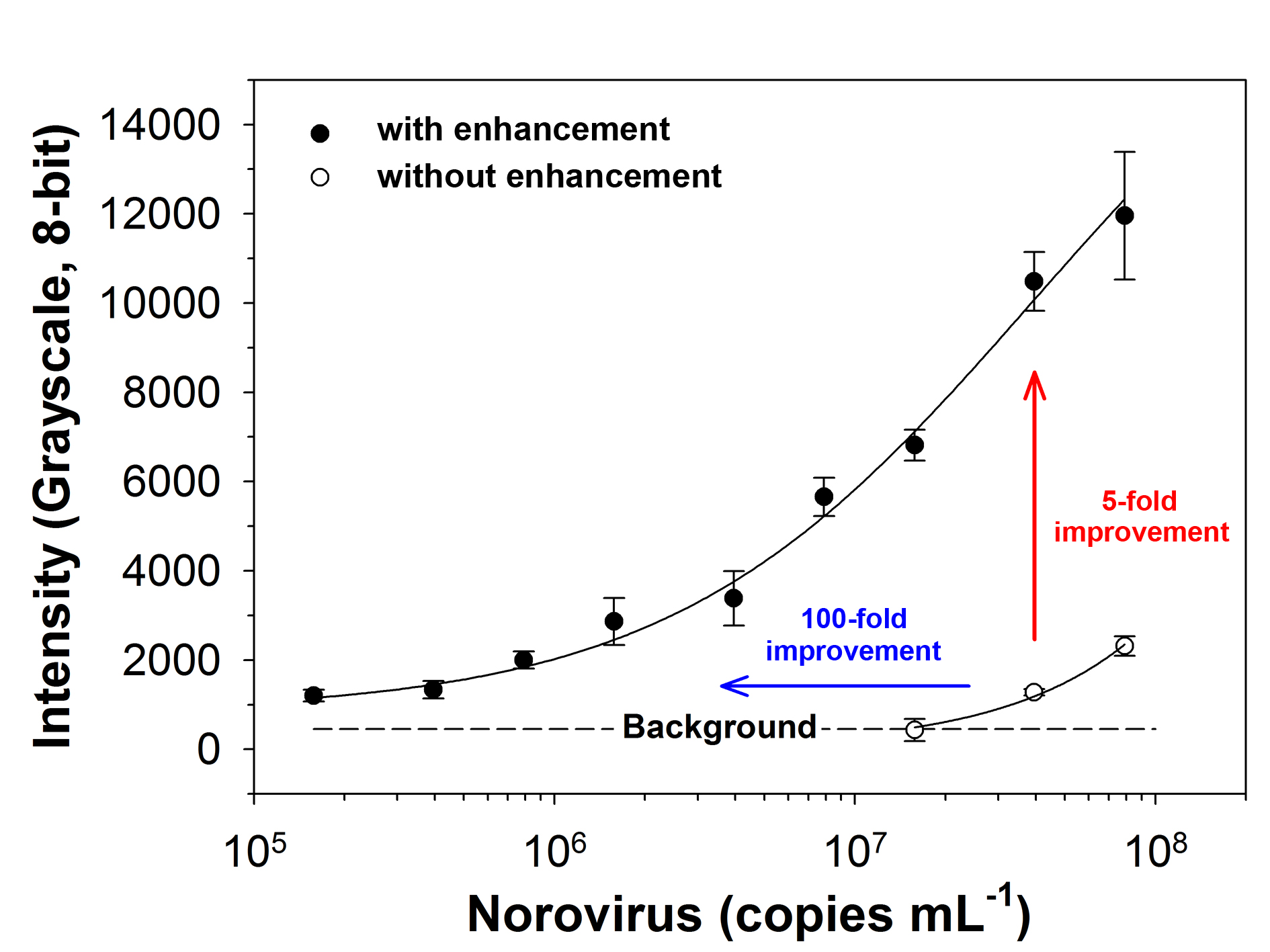


**Figure S4.** Binding curves obtained with/without signal enhancement.


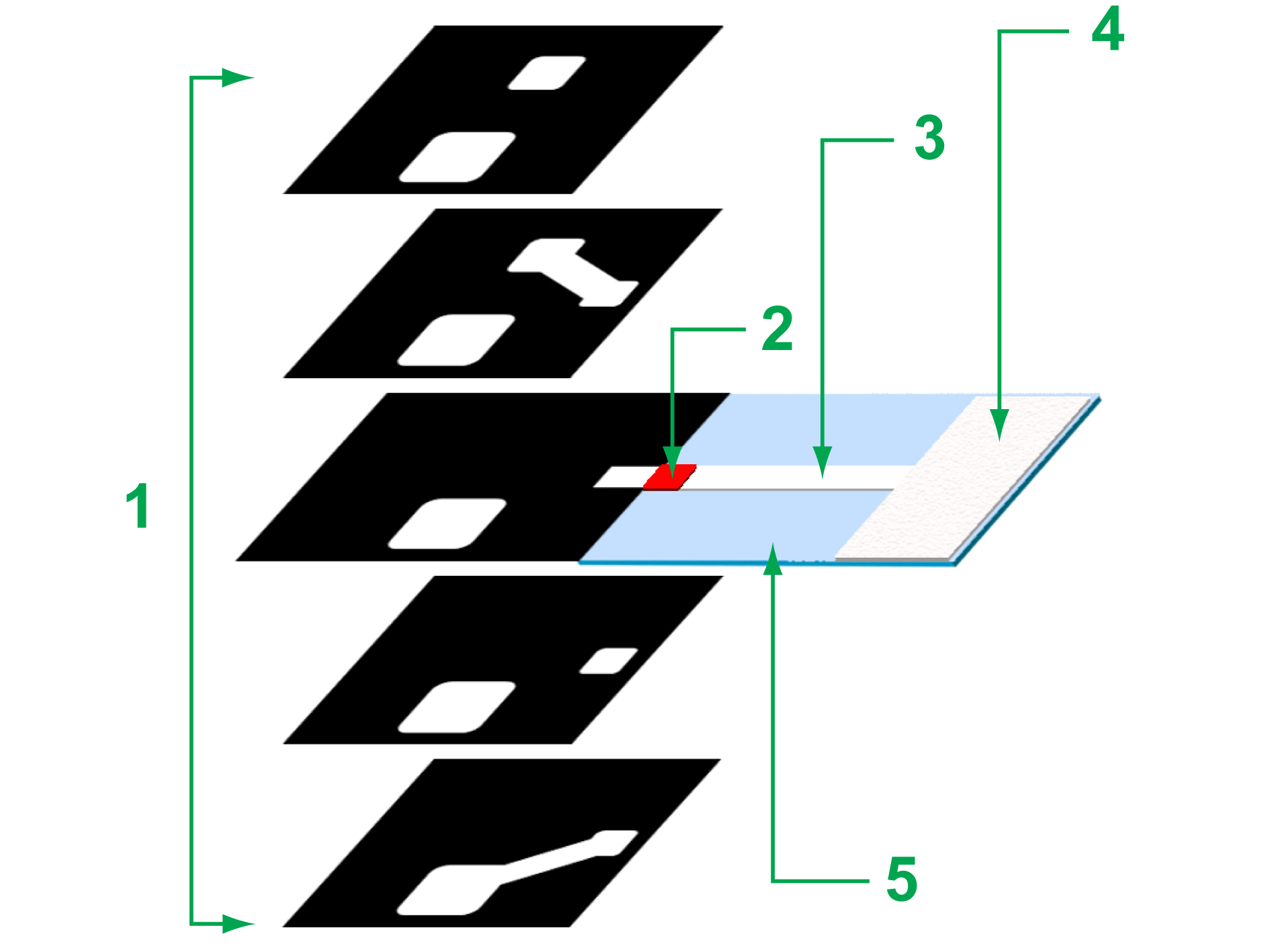


**Figure S5.** Configuration and assembly of 3D slip-PAD components. (1: 3D layers, 2: conjugate pad, 3: nitrocellulose membrane, 4: absorbent pad, 5: adhesive film)

**Movie S1.** Sequential delivery of different fluids in the 3D slip-PAD.

**Movie S2.** Release and mixing of integrated reagents on multiple layers in the 3D slip-PAD.
